# Structural basis for oligomerization of the prokaryotic peptide transporter PepT_So2_

**DOI:** 10.1107/S2053230X19003546

**Published:** 2019-04-24

**Authors:** Reina Nagamura, Masahiro Fukuda, Akihiro Kawamoto, Kyoko Matoba, Naoshi Dohmae, Ryuichiro Ishitani, Junichi Takagi, Osamu Nureki

**Affiliations:** a Department of Biological Sciences, Graduate School of Science, The University of Tokyo, 7-3-1 Hongo, Bunkyo-ku, Tokyo 113-0033, Japan; b Institute for Protein Research, Osaka University, 3-2 Yamadaoka, Suita, Osaka 565-0871, Japan; c RIKEN Advanced Science Institute, 2-1 Hirosawa, Wako-shi, Saitama 351-0198, Japan

**Keywords:** peptide transporter, major facilitator superfamily, lipidic cubic phase, single-particle cryo-electron microscopy, membrane transporter, oligomerization, cryo-EM, X-ray crystallography

## Abstract

Proton-dependent oligopeptide transporters (POTs) belong to the major facilitator superfamily (MFS) and transport dipeptides and tripeptides from the extracellular environment into the target cell. The human POTs PepT1 and PepT2 are also involved in the absorption of various orally ingested drugs. Previously reported structures revealed that the bacterial POTs possess 14 helices, of which H1–H6 and H7–H12 constitute the typical MFS fold and the residual two helices are involved in the cytoplasmic linker. PepT_So2_ from *Shewanella oneidensis* is a unique POT which reportedly assembles as a 200 kDa tetramer. Although the previously reported structures suggested the importance of H12 for tetramer formation, the structural basis for the PepT_So2_-specific oligomerization remains unclear owing to the lack of a high-resolution tetrameric structure. In this study, the expression and purification conditions for tetrameric PepT_So2_ were optimized. A single-particle cryo-EM analysis revealed the tetrameric structure of PepT_So2_ incorporated into Salipro nanoparticles at 4.1 Å resolution. Furthermore, a combination of lipidic cubic phase (LCP) crystallization and an automated data-processing system for multiple microcrystals enabled crystal structures of PepT_So2_ to be determined at resolutions of 3.5 and 3.9 Å. The present structures in a lipid bilayer revealed the detailed mechanism for the tetrameric assembly of PepT_So2_, in which a characteristic extracellular loop (ECL) interacts with two asparagine residues on H12 which were reported to be important for tetramerization and plays an essential role in oligomeric assembly. This study provides valuable insights into the oligomerization mechanism of this MFS-type transporter, which will further pave the way for understanding other oligomeric membrane proteins.

## Abstract 2

Using cryo-EM and X-ray crystallography, the atomic resolution structure of the tetrameric form of the proton-dependent oligopeptide transporter PepT_So2_ was determined and the novel oligomerization mechanism was revealed.

## 1. Introduction and research aim   

Proton-dependent oligopeptide transporters (POTs) belong to the major facilitator superfamily (MFS) and transport short-chain peptides from the extracellular environment into the target cell. The human POTs PepT1 and PepT2 are involved in the absorption of digested peptides and various orally ingested drugs, including antibiotics, antivirals and anticancer agents. Previously reported structures revealed that the bacterial POTs possess 14 helices, of which H1–H6 and H7–H12 constitute the typical MFS fold and the residual two helices, HA and HB, are inserted between H6 and H7 (Doki *et al.*, 2013 ▸; Guettou *et al.*, 2013 ▸; Newstead *et al.*, 2011 ▸; Parker *et al.*, 2017 ▸; Zhao *et al.*, 2014 ▸). PepT_So2_ from *Shewanella oneidensis* is a unique POT transporter that has been reported to assemble as a 200 kDa tetramer in the detergent-solubilized form (Guettou *et al.*, 2013 ▸). Two crystal structures of PepT_So2_ have been reported: tetrameric and dimeric structures determined at resolutions of 4.6 and 3.2 Å, respectively (Guettou *et al.*, 2013 ▸). Although the resolution of the dimeric structure was much higher, the dimer interface was stabilized by a zinc ion contained in the crystallization conditions, suggesting that this dimeric form represents a crystal-packing artifact. Recently, a cryo-electron microscopy (cryo-EM) structure of the PepT_So2_ tetramer embedded in Salipro nanoparticles was reported at 6.5 Å resolution (Frauenfeld *et al.*, 2016 ▸). A structural comparison of the dimeric and tetrameric PepT_So2_ structures suggested that subtle structural changes of H12 and the unconserved Asn468 on H12 are important for tetramer formation (Guettou *et al.*, 2013 ▸). However, the structural basis for the PepT_So2_-specific oligomerization remained unclear owing to the insufficient resolution of the tetrameric structure.

In this study, we overexpressed PepT_So2_ in *Escherichia coli* and optimized the expression conditions. During the expression trials, we found that the number of histidine tags fused to the C-terminus drastically affected the oligomerization state of PepT_So2_. Tetramer formation was confirmed by a single-particle analysis using cryo-EM and was further analyzed by X-ray crystallographic analyses using lipidic cubic phase (LCP) crystallization (Caffrey & Cherezov, 2009 ▸). As a result of the crystallization trials, microcrystals were obtained under multiple conditions. We finally determined structures of PepT_So2_ from two different crystal forms at 3.5 and 3.9 Å resolution by merging small wedge data sets from multiple microcrystals (Yamashita *et al.*, 2018 ▸).

PepT_So2_ forms a tetramer in both of the crystal forms, and the structures superimpose well (with an r.m.s.d. of 0.83 Å for all C^α^ atoms). A slight movement of H12 was also observed, as in the previous structure, suggesting the importance of H12 for tetramer formation. Furthermore, the tetrameric assembly appears to be stabilized by hydrogen bonds between the asparagine residues in H12 and the unconserved extracellular loop (ECL), which was not well resolved in the previously determined low-resolution tetrameric structures (Frauenfeld *et al.*, 2016 ▸; Guettou *et al.*, 2013 ▸). Although the physiological significance of the tetrameric assembly of PepT_So2_ remains unclear, the oligomerization is possibly essential for the stable expression of PepT_So2_ in the plasma membrane. This study provides valuable insights into the oligomerization mechanism of this MFS-type transporter, which will further facilitate the understanding of other oligomeric membrane proteins.

## 2. Materials and methods   

### 2.1. Macromolecule production   

The gene encoding full-length PepT_So2_ (UniProt Q8EHE6) was subcloned into a pET-modified vector (Nishizawa *et al.*, 2013 ▸) which contains the DNA sequence encoding the Tobacco etch virus (TEV) protease cleavage site (ENLYFQG) followed by a His_6_ or His_8_ tag at the 3′-terminus. The macromolecule-production information is summarized in Table 1 ▸. All of the PepT_So2_ mutants were produced by a PCR-based site-directed mutagenesis method using PrimeSTAR Max DNA polymerase (Takara).

### 2.2. Optimization of the expression conditions for PepT_So2_   

The plasmid was introduced into *Escherichia coli* Rosetta 2 (DE3) cells (Novagen) or genetically modified C41 (DE3) cells harboring the pRARE plasmids encoding the tRNAs for codons that are rarely used in *E. coli*. The transformed cells were grown in 2.5 ml Luria–Bertani (LB) medium containing 50 µg ml^−1^ ampicillin with or without 0.4% glucose or in 2.5 ml Terrific Broth (TB) at 310 K. When the absorbance at 600 nm (*A*_600_) reached 0.5–0.8, protein expression was induced with 0.5 m*M* isopropyl β-d-1-thio­galactopyranoside (IPTG) and the cells were grown for about 18 h at 293 K. The cells were centrifuged at 8000g for 1 min and were then suspended in solubilization buffer [50 m*M* Tris–HCl pH 8.0, 300 m*M* NaCl, 15 m*M* imidazole, 0.5 m*M* Tris(2-carboxyethyl)phos­phine (TCEP), 10% glycerol, 2% *n*-dodecyl-β-d-maltoside (DDM)] supplemented with cOmplete EDTA-free Protease Inhibitor Cocktail (Roche). The cells were disrupted by sonication using a Bioruptor UCW-310 (Cosmo Bio) and solubilized by gentle rotation at 277 K for 1.5 h. After the removal of unsolubilized materials by ultracentrifugation at 104 000*g* for 30 min at 277 K, the supernatant was mixed with the P3NTA probe (produced in-house; Backmark *et al.*, 2013 ▸) and analyzed by fluorescence-detection size-exclusion chromatography (FSEC; Kawate & Gouaux, 2006 ▸).

### 2.3. Large-scale expression and purification of PepT_So2_   

The plasmid containing C-terminally His_6_-tag-fused PepT_So2_ was introduced into *E. coli* Rosetta 2 (DE3) cells (Novagen). The transformed cells were grown in 18 l LB medium containing 50 µg ml^−1^ ampicillin at 310 K. When the *A*_600_ reached 0.5–0.8, expression was induced with 0.5 m*M* IPTG and the cells were grown for about 18 h at 293 K. The cells were suspended in phosphate-buffered saline. The suspension was homogenized and mixed on ice, and was disrupted by 4–5 passes at 130 MPa using a Microfluidizer (Microfluidics). After centrifugation at 25 000*g* for 20 min to remove the debris, the supernatant was ultracentrifuged at 125 000*g* for 1 h at 277 K using a Type 45 Ti rotor (Beckman Coulter). The membrane fraction was resuspended in membrane buffer (50 m*M* Tris–HCl pH 8.0, 300 m*M* NaCl, 0.5 m*M* TCEP, 10% glycerol) supplemented with cOmplete EDTA-free Protease Inhibitor Cocktail (Roche) and stored at 193 K until use.

The membrane fraction was solubilized in solubilization buffer supplemented with cOmplete EDTA-free Protease Inhibitor Cocktail (Roche) at 277 K for 2 h. After removal of unsolubilized material by ultracentrifugation at 125 000*g* for 30 min at 277 K using a Type 45 Ti rotor (Beckman Coulter), the supernatant was mixed with 25 ml Ni–NTA Superflow resin (Qiagen) equilibrated with Ni buffer (50 m*M* Tris–HCl pH 8.0, 300 m*M* NaCl, 0.5 m*M* TCEP, 5% glycerol, 0.05% DDM) containing 15 m*M* imidazole for 1 h at 277 K. The mixture was transferred into an Econo-column (Bio-Rad) and the flowthrough fraction was collected. The resin was washed with ten column volumes of Ni buffer containing 50 m*M* imidazole, and the protein sample was eluted with Ni buffer containing 300 m*M* imidazole. To cleave the His_6_ tag that was fused to the C-terminus of PepT_So2_, His-tagged TEV protease (produced in-house) was added to the eluted fraction to a 1:75(*w*:*w*) protease:protein ratio and the solution was dialyzed twice against 1 l dialysis buffer (50 m*M* Tris–HCl pH 8.0, 300 m*M* NaCl, 0.25 m*M* TCEP, 5% glycerol, 0.03% DDM). To remove the cleaved His tag and His-tagged TEV protease, the solution was mixed with Ni–NTA resin equilibrated with dialysis buffer for 1.5 h at 277 K and the collected flowthrough fraction was concentrated using an Amicon Ultra centrifugal filter (100 kDa molecular-weight cutoff; Millipore). The concentrated sample was ultracentrifuged at 40 000 rev min^−1^ for 20 min using an S55A2 rotor (Hitachi Koki) and the supernatant was applied onto a HiLoad 16/600 Superdex 200 pg column (GE Healthcare) equilibrated with SEC buffer consisting of 50 m*M* Tris–HCl pH 8.0, 300 m*M* NaCl, 5% glycerol, 0.03% DDM. The purity and homogeneity of the protein sample were assessed by FSEC. The purity of the protein sample was also assessed by SDS–PAGE. The protein concentrations were estimated based on the absorbance at 280 nm (*A*_280_) as measured using a NanoDrop spectrophoto­meter (Thermo Fisher Scientific). The protein bands detected in the SDS–PAGE gel were analyzed using the peptide mass fingerprint method.

### 2.4. Expression and purification of saposin A   

The coding region of saposin A was cloned into a modified pET-28b vector that harbors an N-terminal His_6_ tag followed by the ‘eTEV’ sequence (Tabata *et al.*, 2018 ▸), resulting in the protein sequence MGSSHHHHHHSSGLVPRENLYFQGMGSLPCDICKDVVTAAGDMLKDNATEEEILVYLEKTCDWLPKPNMSASCKEIVDSYLPVILDIIKGEMSRPGEVCSALNLCES. The protein cleaved by TEV protease represents the saposin A polypeptide sequence with three additional N-terminal residues (Gly-Met-Gly). The expression and purification of saposin A were performed according to a previously reported method (Frauenfeld *et al.*, 2016 ▸).

### 2.5. Reconstruction of PepT_So2_ into Salipro particles   

For the reconstruction of PepT_So2_ into Salipro nanoparticles, 8 µl of purified PepT_So2_ at 20.4 mg ml^−1^ in TBSGD buffer (25 m*M* Tris–HCl pH 8.0, 5% glycerol, 0.03% DDM), 30 µl of a 5 mg ml^−1^ DMPG (Avanti) lipid solution in lipid-solubilization buffer (50 m*M* HEPES–NaOH pH 7.5, 150 m*M* NaCl, 0.224% DDM) and 12 µl HBSD buffer (50 m*M* HEPES–NaOH pH 7.5, 150 m*M* NaCl, 0.03% DDM) were mixed and incubated at 298 K for 5 min. Next, 120 µl of purified saposin A at 1.2 mg ml^−1^ was added and the mixture was incubated at 278 K for 5 min. Subsequently, 114 µl saposin SEC buffer, consisting of 25 m*M* sodium phosphate pH 7.4, 200 m*M* NaCl, was added to the mixture and incubated at 278 K for 5 min, after which 234 µl saposin SEC buffer was added. The mixture was injected into a Superdex 200 Increase 10/300 GL column (GE Healthcare) equilibrated with saposin SEC buffer. Fractions containing Salipro–PepT_So2_ were pooled and concentrated to ∼0.5 mg ml^−1^ using an Amicon Ultra centrifugal filter (30 kDa molecular-weight cutoff; Millipore).

### 2.6. Cryo-EM sample preparation and data collection   

Quantifoil copper 200 mesh R1.2/1.3 holey carbon grids were glow-discharged on a glass slide for 30 s. A 2.6 µl aliquot of the sample solution was applied onto the grid and blotted by filter paper for 4.5 s at 100% humidity and 277 K, and the grid was then quickly frozen by rapidly plunging it into liquid ethane using a Vitrobot Mark IV (Thermo Fisher Scientific). The grid was inserted into a Talos Arctica FEG transmission electron microscope (Thermo Fisher Scientific) operated at 200 kV, with the cryo-specimen stage cooled with liquid nitrogen. Cryo-EM images were recorded using a Falcon 3EC 4k × 4k CMOS direct electron detector (Thermo Fisher Scientific) in counting mode at a nominal magnification of ×96 000, corresponding to an image pixel size of 0.87 Å, using the *EPU* software package. Movie frames of the PepT_So2_ tetramer embedded in Salipro were collected from 30° to 50° tilts of the stage in 10° increments at a dose rate of 1.2 e per pixel per second and an exposure time of 68.38 s. The total accumulated exposure of 82 e Å^−2^ was fractionated into 102 frames. A data set of 1609 micrographs (30° tilt, 1275 micrographs; 40° tilt, 272 micrographs; 50° tilt, 62 micrographs) was collected in a single session using a defocus range between 1.0 and 3.0 µm.

### 2.7. Image processing   

Data sets from the various tilts from 30° to 50° were used. The movie frames were subsequently aligned to correct for beam-induced movement and drift using *MotionCor*2 (Zheng *et al.*, 2017 ▸), and the parameters for the contrast transfer function (CTF) were estimated using *Gctf* (Zhang, 2016 ▸). A total of 288 417 particle images were automatically picked from 1484 micrographs using *Gautomatch* (http://www.mrc-lmb.cam.ac.uk/kzhang/), and two-dimensional (2D) and three-dimensional (3D) classifications were then performed using *Relion*-3.0 (Zivanov *et al.*, 2018 ▸). Particle images from the good 2D class were selected to obtain the initial 3D model of the PepT_So2_ tetramer embedded in Salipro using *cryoSPARC* (Punjani *et al.*, 2017 ▸) with *C*4 symmetry. A total of 43 172 particles from the best 3D class were subjected to 3D refinement, which produced a reconstruction with a resolution of 5.9 Å and a *B* factor of −307 Å^2^. The 3D-refined structure was further refined with per-particle defocus with a search range of ±2000 Å and Bayesian polishing, which improved the resolution to 4.3 Å with a *B* factor of −171 Å^2^. To improve the resolution further, CTF refinement with beam-tilt correction and Bayesian polishing was iterated two times, giving 4.3 Å resolution and a *B* factor of −161 Å^2^ and finally 4.1 Å resolution and a *B* factor of −147 Å^2^. The 3D density map was visualized using *UCSF Chimera* (Pettersen *et al.*, 2004 ▸).

### 2.8. X-ray crystallographic analyses   

The PepT_So2_ sample was mixed with monoolein (Nu-Chek Prep) at a 2:3(*w*:*w*) protein:lipid ratio using coupled syringes. Drops of the mixture (30 nl) were dispensed onto 96-well Laminex glass sandwich plates (MD11-50-100; Molecular Dimensions) and overlaid with 800 nl reservoir solution using a Gryphon LCP crystallization robot (Art Robbins). The initial crystallization screening was performed at 293 K using in-house-produced grid-screening crystallization kits as reservoir solutions. To optimize the crystallization conditions, StockOptions Salt and Additive Screen (Hampton Research) was added to the reservoir solutions for LCP crystallization, in addition to optimization of the pH and the concentrations of precipitants and salts. Crystals were picked up using MicroMeshes (MiTeGen), MicroMounts (MiTeGen) or LithoLoops (Protein Wave) and were flash-cooled in liquid nitrogen. Crystallization information is provided in Table 2 ▸.

### 2.9. X-ray data collection and processing   

All X-ray diffraction data sets were collected by the helical data-collection method using the microfocus beam at SPring-8 beamline BL32XU (Hirata *et al.*, 2013 ▸). The locations of well diffracting crystals were identified by raster scanning, and a 5° or 10° wedge of data was collected from each crystal. All diffraction data were processed with *XDS* (Kabsch, 2010 ▸) and were merged with *XSCALE* based on hierarchical clustering analysis with the cross-correlation method implemented in the *KAMO* software (Yamashita *et al.*, 2018 ▸).

Molecular replacement was performed with *Phaser* (McCoy *et al.*, 2007 ▸). For structure determination of the form *B* crystal, the structure of molecule *A* of PepT_So2_ (PDB entry 4lep), reported as a dimeric form, was used as the search model. For structure determination of the form *A* crystal, the structure determined from the form *B* crystal (PDB entry 6jkc) was used as the search model. Model building was performed with *Coot* (Emsley & Cowtan, 2004 ▸; Emsley *et al.*, 2010 ▸). Refinement was performed with *phenix.refine* (Adams *et al.*, 2010 ▸) and *REFMAC*5 (Murshudov *et al.*, 2011 ▸) using the *CCP*4*i*2 interface (Potterton *et al.*, 2018 ▸). The data-collection statistics are summarized in Table 3 ▸. Molecular graphics were generated using *CueMol* (http://www.cuemol.org).

### 2.10. Model building for cryo-EM   

The crystal structure of PepT_So2_ determined in this work (form *B* crystal) was fitted as a rigid body into the 3D density map using *UCSF Chimera* and *Coot* (Emsley & Cowtan, 2004 ▸; Emsley *et al.*, 2010 ▸). Refinement was performed with *phenix.refine* (Adams *et al.*, 2010 ▸). The conformations were refined using real-space refinement in *PHENIX* (Adams *et al.*, 2010 ▸) with secondary-structure restraints. We first performed model refinement for each subunit separately against the corresponding EM maps. To resolve the possible clashes in the subunit interfaces, we refined the entire models against the corresponding EM maps. Table 4 ▸ summarizes the refinement statistics for the overall structure, the deposited maps and their associated coordinates.

## 3. Results   

### 3.1. FSEC-based optimization of expression conditions   

**Figure 1:**
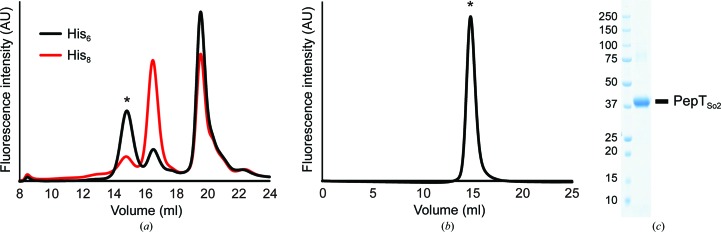
Protein preparation. (*a*) Fluorescence-detection size-exclusion chromatography (FSEC)-based analysis of His_6_ (black) and His_8_ (red) constructs. The histidine tag-specific fluorescent probe P3NTA was used for detection (excitation, 482 nm; emission, 520 nm). The peak corresponding to tetrameric PepT_So2_ is marked with an asterisk. (*b*) SEC chromatogram of purified PepT_So2_. The fluorescent signals, which were mainly derived from the tryptophan residues, were monitored by the fluorescence detector, with excitation at 280 nm and emission at 350 nm. The peak corresponding to tetrameric PepT_So2_ is marked with an asterisk. (*c*) SDS–PAGE analysis with Coomassie Brilliant Blue staining. Left lane, molecular-weight markers (labeled in kDa); right lane, the merged peak fraction from the SEC purification.

For structural analysis of tetrameric PepT_So2_, the expression and purification conditions were optimized. Full-length PepT_So2_ fused with a His tag at the C-terminus was expressed using a modified pET vector (Nishizawa *et al.*, 2013 ▸; Table 1 ▸), and the expression level and homogeneity of the samples were evaluated by an FSEC-based high-throughput screening method (Kawate & Gouaux, 2006 ▸) using the P3NTA peptide as a probe (Backmark *et al.*, 2013 ▸). Unexpectedly, the number of histidine residues fused to the C-terminus drastically affected the oligomerization state of PepT_So2_, and the His_6_-tagged PepT_So2_ formed a tetramer more readily (Fig. 1 ▸ *a*). Under the conditions that we tested, the expression levels of PepT_So2_ were greater in the BL21 (DE3) derivative strain of *E. coli* compared with the C41 (DE3) strain, which was used in the previous studies (Guettou *et al.*, 2013 ▸). The presence or absence of tRNAs reading rare codons in the expression host did not affect the expression level. The addition of glucose to the medium did not affect the expression level. The expression level of PepT_So2_ was higher when using LB medium compared with the TB medium used in the previous study. These culture conditions affected the expression level but not the oligomerization state of PepT_So2_.

### 3.2. Large-scale expression and purification of PepT_So2_   

C-terminally His_6_-tagged PepT_So2_ was expressed on a large scale under the conditions optimized in this study. PepT_So2_ solubilized with DDM was purified by Ni–NTA affinity chromatography followed by cleavage of the His tag, a second Ni–NTA chromatography step to remove the His-tagged TEV protease and size-exclusion chromatography (SEC). The SEC chromatogram peak showed monodispersity, and the peak fractions showed a single band in the SDS–PAGE analysis, demonstrating high purity and homogeneity of the obtained PepT_So2_ sample (Figs. 1 ▸ *b* and 1 ▸ *c*). The elution volume of PepT_So2_ in the SEC chromatogram was 14.8 ml in DDM-containing buffer using a Superose 6 Increase column (GE Healthcare) (Fig. 1 ▸ *b*), which was a reasonable elution position for tetrameric PepT_So2_ (the molecular weight is about 200 kDa). The final yield was about 2.5 mg per litre of *E. coli* culture.

### 3.3. Single-particle cryo-EM analyses of PepT_So2_   

**Figure 2:**
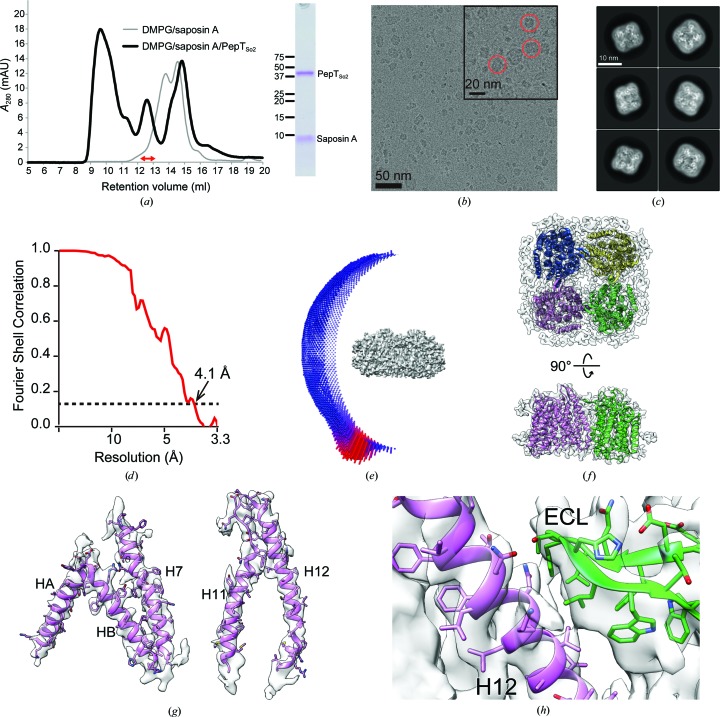
Single-particle analysis of PepT_So2_ using cryo-electron microscopy. (*a*) Salipro reconstitution of PepT_So2_. SEC chromatograms before and after reconstitution of PepT_So2_ are shown as gray and black lines, respectively (left panel). The peak fractions indicated by the red arrow were analyzed by SDS–PAGE (right panel). (*b*) Raw electron micrograph of PepT_So2_. The Salipro-reconstituted PepT_So2_ particles are indicated by red circles. (*c*) Representative 2D class averages generated from the particles. The scale bar represents 10 nm. (*d*) Gold-standard FSC between two independently refined half-maps. (*e*) Euler angle distribution of all particles collected by the tilting method. (*f*) 3D density maps of the tetrameric PepT_So2_ incorporated in the Salipro nanoparticle. The atomic model was refined by using the crystal structure determined in this study as the initial model. (*g*) Densities of selected helices and side chains. (*h*) The density around the inter-protomer interaction area. The extracellular loop of a protomer is colored green and H12 of the neighboring protomer is colored magenta.

To gain structural insight into oligomer formation by PepT_So2_, we reconstructed PepT_So2_ into Salipro nanoparticles using saposin A and DMPG lipids, and performed single-particle analysis using cryo-electron microscopy (Figs. 2 ▸ *a*–2 ▸ *e*, Supplementary Fig. S1). Although square-shaped particles were clearly observed in the cryo-EM micrographs of PepT_So2_ reconstructed in Salipro, the overall resolution was limited to 6.7 Å and the quality of the map was too poor to refine the atomic model owing to severe orientation bias (Supplementary Fig. S1). Interestingly, this preferred specimen-orientation problem was not reported in the previous study (Frauenfeld *et al.*, 2016 ▸). In the previous study, bovine brain lipid extract was used for reconstruction, but this product is no longer commercially accessible. The difference in the lipids used for reconstruction in this study might affect the physical properties of the specimen under solution conditions and thus cause the preferred specimen-orientation problem. To alleviate the orientation bias, we tried to collect data by tilting the grid from 30° to 50° (Tan *et al.*, 2017 ▸), which drastically improved the data quality (Supplementary Fig. S1). A total of 43 172 selected particles from three different tilt angles (30°, 40° and 50°) yielded a 3D EM map at an overall resolution of 4.1 Å, according to the gold-standard Fourier shell correlation (FSC) 0.143 criterion (Scheres & Chen, 2012 ▸; Fig. 2 ▸ *d*, Supplementary Fig. S2, Table 4 ▸). The tetrameric organization of PepT_So2_ is clearly visualized in the final 3D density map (Fig. 2 ▸ *f*), in which the secondary-structural features and the side-chain densities from bulky amino-acid residues are clearer compared with the previous study (6.5 Å resolution; Frauenfeld *et al.*, 2016 ▸; Fig. 2 ▸ *g*). Residual density derived from the Salipro scaffold composed of saposin A and lipids was also observed around the tetrameric PepT_So2_ molecule; however, the density in these regions was not so clear compared with the PepT_So2_ molecule (Fig. 2 ▸ *h*), suggesting flexibility of the Salipro scaffold region. The EM density map suggested a contribution of the extracellular loops between the TM helices that were not well resolved in the previous study to the tetramer assembly (Figs. 2 ▸ *h* and 2 ▸ *f*). However, the atomic-level oligomerization mechanism remained unclear because of the resolution limit.

### 3.4. X-ray crystallographic analyses of PepT_So2_   

**Figure 3:**
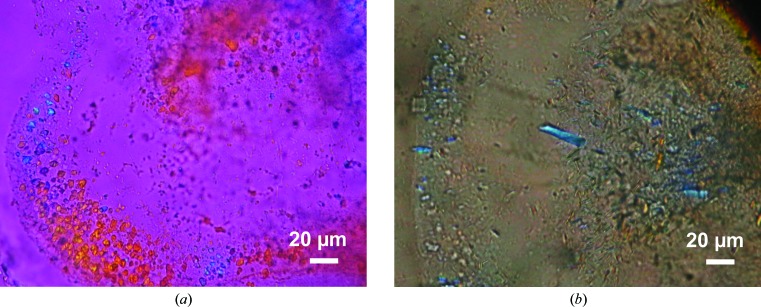
LCP crystals of PepT_So2_. (*a*) Crystals of form *A*. (*b*) Crystals of form *B*.

To further improve the resolution, we tried to crystallize tetrameric PepT_So2_ using the LCP method (Caffrey & Cherezov, 2009 ▸) at protein concentrations of 20, 45 and 75 mg ml^−1^. Microcrystals of PepT_So2_ were obtained using a 45 mg ml^−1^ sample under multiple conditions containing PEG 200 as a precipitant (Fig. 3 ▸). Since the size of the crystals was too small and data collection from a single crystal was impossible, small wedge data were collected from microcrystals using the microfocus beam at BL32XU at SPring-8, Hyogo, Japan and were combined using the multi-crystal merging software *KAMO* (Yamashita *et al.*, 2018 ▸). By using the molecular-replacement method with the previously reported structure of the PepT_So2_ dimer (PDB entry 4lep, molecule *A*) as the search model (Guettou *et al.*, 2014 ▸), the structures of the form *A* and *B* crystals were finally determined at resolutions of 3.9 and 3.5 Å, respectively (Table 3 ▸).

### 3.5. Overall structure of tetrameric PepT_So2_   

**Figure 4:**
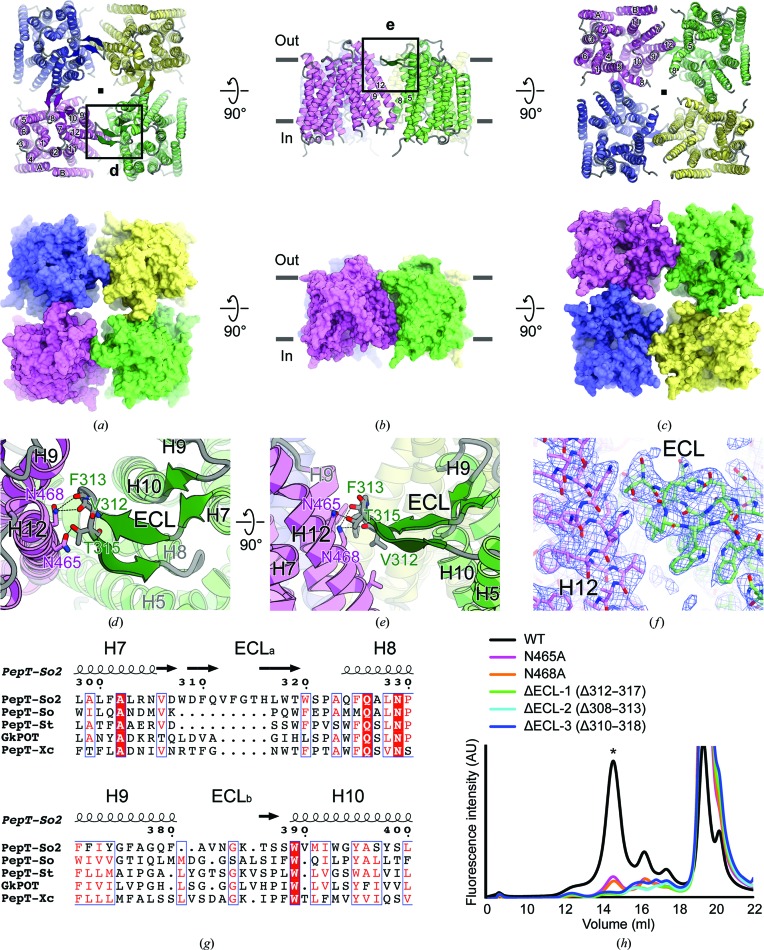
Tetrameric organization of PepT_So2_. (*a*, *b*, *c*) The overall structure of tetrameric PepT_So2_ is viewed from the (*a*) extracellular, (*b*) membrane and (*c*) intracellular sides. The four protomers are colored magenta, green, blue and yellow. The rectangles labeled ‘**d**’ and ‘**e**’ indicate the regions highlighted in (*d*) and (*e*), respectively. (*d*, *e*) The intermolecular interactions of the extracellular loop (ECL) with H12 of the neighboring protomer are viewed from the (*d*) extracellular and (*e*) membrane sides. The possible hydrogen bonds of up to 3.2 Å are shown as black dotted lines. (*f*) A 2*mF*_o_ − *DF*_c_ electron-density map of the interacting area between the ECL and H12 contoured at 1.1σ. The C atoms of the two protomers are colored magenta and green. (*g*) Sequence alignment of PepT_So2_ with other prokaryotic POT families in the ECL area. (*h*) FSEC-based mutational analyses of PepT_So2_. The peak corresponding to tetrameric PepT_So2_ is marked with an asterisk.

The PepT_So2_ structures obtained from the two crystal forms (forms *A* and *B*) were assembled as tetramers in similar manners (r.m.s.d. of 0.83 Å for all C^α^ atoms), although the space groups and the crystal packings were different (Table 3 ▸, Supplementary Fig. S3). Thus, in the following we focus on the structure obtained from the form *B* crystal (Figs. 4 ▸ *a*, 4 ▸ *b* and 4 ▸ *c*), as the quality of its electron density is better than that of the other crystal form. All of the protomers in the tetramer adopted the inward-open conformation, in which a large central hydrophilic substrate-binding pocket faces the intracellular side of the membrane. The structural comparison shows that the present tetrameric structure superimposes well with the previously reported dimeric structure at the protomer level (r.m.s.d. of 1.1 Å for all C^α^ atoms; Supplementary Fig. S4*a*). The present crystal structures fitted well to the cryo-EM map obtained in this study (Fig. 2 ▸ *f*, Supplementary Fig. 4*b*).

### 3.6. Intermolecular interactions of the PepT_So2_ tetramer   

In the structure determined in this study, a sliding motion of H12 was also observed compared with the previously reported dimeric structure (Supplementary Fig. 4*a*), supporting the importance of H12 for tetramer formation suggested in the previous report (Guettou *et al.*, 2014 ▸). On the intracellular side, inter-subunit hydrophobic interactions were formed between H12 of one protomer and H5 and H8 of the neighboring protomer (Fig. 4 ▸ *c*). On the extracellular side of the present structure, electron density for the extracellular loop (ECL), which was not well resolved in the previous structures, was clearly observed (Fig. 4 ▸ *f*). The ECL consists of two extracellular linkers between H7–H8 (ECL_a_) and H9–H10 (ECL_b_) (Figs. 4 ▸ *d* and 4 ▸ *e*), in which four β-strands form parallel and antiparallel β-sheet structures. The structure suggested that the ECL plays an important role in PepT_So2_ tetramer formation, together with H12 (Figs. 4 ▸ *d* and 4 ▸ *e*). The ECL interacts with H9 and H12 of the neighboring protomer by hydrophobic or hydrogen-bond interactions (Figs. 4 ▸ *a*, 4 ▸ *d* and 4 ▸ *e*). In the previous study, the importance of Asn468 in H12 for the tetramerization of PepT_So2_ was suggested (Guettou *et al.*, 2014 ▸). The higher resolution structure determined in this study clearly shows that the side chains of Asn465 and Asn468 in H12 form hydrogen bonds to the ECL of the neighboring protomer, thus stabilizing the tetramer (Figs. 4 ▸ *a*, 4 ▸ *d* and 4 ▸ *e*). These two asparagine residues and the amino-acid sequence of the ECL are not conserved among the POT family members. Interestingly, the ECL of PepT_So2_ is longer compared with those of other prokaryotic orthologues with structures that were reported to be monomers or dimers (Fig. 4 ▸ *g*). In contrast, among the POT family members that are phylogenetically closer to PepT_So2_ at the amino-acid level, the peptide lengths of the ECL are preserved, while the amino-acid sequences are not highly conserved. These results suggest that these non­conserved amino-acid residues play an important role in PepT_So2_-specific tetramer formation, which is further supported by the mutational analyses using FSEC (Fig. 4 ▸ *h*).

## 4. Discussion   

Many membrane proteins exist and function as oligomers in the membrane. The oligomeric states are highly diverse among the individual membrane proteins, and are sometimes in equilibrium depending on the protein concentration in the membrane. For membrane transporters, it has been suggested that oligomerization plays various roles, such as in transport activity, membrane trafficking, regulation of function and turnover (Alguel *et al.*, 2016 ▸). Oligomerization can allow transporters to form a stable functional structure. In some cases, such as ATP-binding cassette (ABC) transporters (Locher, 2016 ▸) and the small multidrug transporter EmrE (Chen *et al.*, 2007 ▸), the interface of the protomers forms the substrate-binding site and translocation pathway. Another example is the well studied bacterial glutamine transporter Glt_ph_, which assembles as a trimer regardless of concentration, although each protomer contains the substrate-transport pathway (Yernool *et al.*, 2004 ▸). Previous functional and structural analyses have suggested that the trimeric arrangement of Glt_ph_ plays a critical role in conformational changes during the transport cycle (Reyes *et al.*, 2009 ▸). In the above cases, the manner of oligomeric arrangement is widely preserved at the family level and is critical for proper function.

In contrast, there are some membrane transporters that form species-specific or subtype-specific oligomers, in which each protomer consists of a transport unit. This study provides structural insight into one of these types of transporters, PepT_So2_, which belongs to the POT family of the MFS. PepT_So2_ is derived from the Gram-negative anaerobic bacterium *S. oneidensis* and is known to assemble as a tetramer (Guettou *et al.*, 2014 ▸). In contrast, another POT family protein from *S. oneidensis*, PepT_So_, exists as a monomer in solution (Guettou *et al.*, 2014 ▸). Previous reports also revealed that other POT family members exist as mixtures of monomers and dimers, while tetramer formation has only been observed for PepT_So2_ (Guettou *et al.*, 2014 ▸; Löw *et al.*, 2013 ▸). In this study, a combination of X-ray crystallographic and single-particle cryo-EM analyses revealed the species-specific tetramer formation mechanism of PepT_So2_, in which the two asparagine residues in H12 and the unpreserved ECL play key roles. Although the physiological significance of tetramer formation remains unknown, tetramer formation may contribute to stable expression and/or membrane local­ization. In all of the structures determined in this study, each PepT_So2_ protomer adopts a similar inward-open state, which might reflect cooperative substrate transport in the tetramer. It is also possible that the oligomerization state of PepT_So2_ under physiological conditions is regulated by lipid molecules in the membrane, an unknown partner protein or the binding of substrates or additional cofactors. However, the molecular basis and the physiological significance of the oligomerization of membrane transporters, including PepT_So2_, remain unclear at many points and further research is awaited.

## 5. Data availability   

Data supporting the findings of this manuscript are available from the corresponding authors upon reasonable request. The coordinates and structure factors from the X-ray crystallo­graphic analyses have been deposited in the Protein Data Bank (PDB) with accession codes 6jkd and 6jkc for the form *A* (space group *I*4) and form *B* (space group *P*42_1_2) crystals, respectively. X-ray diffraction images are also available from the Zenodo data repository (https://zenodo.org/record/2533841). The coordinates used for electron-microscopic analysis have been deposited in the PDB with accession code 6ji1. The EM map of PepT_So2_ embedded in Salipro nanoparticles has been deposited in the Electron Microscopy Data Bank (EMDB) under accession code EMD-9832.

## Supplementary Material

PDB reference: PepT_So2_, cryo-EM structure, 6ji1

PDB reference: X-ray structures, 6jkc

PDB reference: 6jkd

Supplementary Figures.. DOI: 10.1107/S2053230X19003546/tb5139sup1.pdf

## Figures and Tables

**Table 1:** Macromolecule-production information

Source organism	*S. oneidensis*
DNA source	UniProt Q8EHE6
Expression vector	Modified pET-28a
Expression host	*E. coli* Rosetta 2 (DE3)
Complete amino-acid sequence of the construct produced†	MTLGTNQVSKTHSFMTVSLIELWERFGYYGMQALIVYFMVQRLGFDDSRANLVWSACAALIYVSPAIGGWVGDKILGTKRTMLLGAGILSVGYALMTVPTENTWFMFSALGVIVVGNGLFKPNAGNLVRKIYEGDDSKIDSAFTIYYMAVNVGSTFSMLLTPWIKDYVNAQYGNEFGWHAAFAVCCVGILVGLGNYALMHKSLANYGSEPDTRPVNKKSLAIVLALAALSVVASAIILEYEDVARVFVYAAGVAVLGIFFHLIRTSEPSERAGLIAALILTVQTVFFFIFYQQMSTSLALFALRNVDWDFQVFGTHLWTWSPAQFQALNPIWIMVLSPVLAWSYSWAGRNNKDFSIAAKFALGFAVVAIGFFIYGFAGQFAVNGKTSSWVMIWGYASYSLGELLVSGLGLAMIARYVPARMGGFMMGAYFVASGISQYLGGVVANFASVPQDLVDPLQTLPVYTNLFNKLGVAAVVCTIIALAVLPLMRRLTESHHAHSSIENNAAASLRDVKAEQLESSGENLYFQ(GQFTSSVHHHHHH)

† The cloning artifacts are underlined. The residues cleaved by TEV protease are in parentheses.

**Table 2:** Crystallization conditions

	Form *A* crystal	Form *B* crystal
Method	Lipidic cubic phase (LCP)	Lipidic cubic phase (LCP)
Plate type	96-well glass sandwich plate	96-well glass sandwich plate
Temperature (K)	293	293
Protein concentration (mg ml^−1^)	45	45
Buffer composition of protein solution	300 m*M* NaCl, 50 m*M* Tris–HCl pH 8.0, 5% glycerol, 0.03% DDM	300 m*M* NaCl, 50 m*M* Tris–HCl pH 8.0, 5% glycerol, 0.03% DDM
Composition of reservoir solution	40% PEG 200, 100 m*M* sodium malonate pH 7.0, 100 m*M* sodium acetate pH 4.0	40% PEG 200, 100 m*M* NaCl, 100 m*M* sodium acetate pH 4.0
Volume of LCP drop (nl)	30	30
Volume of reservoir (nl)	800	800

**Table 3:** X-ray data-collection and refinement statistics Values in parentheses are for the outer shell.

	Form *A* crystal (PDB entry 6jkd)	Form *B* crystal (PDB entry 6jkc)
X-ray data collection		
Diffraction source	BL32XU, SPring-8	BL32XU, SPring-8
Wavelength (Å)	1.0000	1.0000
Temperature (K)	100	100
Detector	EIGER X 9M, Dectris	EIGER X 9M, Dectris
Crystal-to-detector distance (mm)	300	280
Rotation range per image (°)	0.1	0.1
Rotation range per crystal (°)	10	5
No. of crystals	10	9
Space group	*I*4	*P*42_1_2
*a*, *b*, *c* (Å)	115.1, 115.1, 110.1	119.4, 119.4, 104.3
Resolution range (Å)	50–3.90 (4.14–3.90)	47.53–3.50 (3.71–3.50)
Total No. of reflections	23358	29586
No. of unique reflections	6448	8312
Completeness (%)	97.4 (97.8)	83.0 (83.4)
Multiplicity	3.6 (3.7)	3.6 (3.5)
〈*I*/σ(*I*)〉	4.38 (1.44)	5.49 (1.22)
*R*_meas_	0.455 (1.277)	0.328 (1.586)
CC_1/2_	0.946 (0.459)	0.961 (0.293)
Refinement		
Resolution (Å)	46.65–3.90	47.53–3.50
*R*_work_/*R*_free_	0.2524/0.2832	0.2672/0.3030
No. of atoms		
Protein	3476	3498
Ligand	0	0
Solvent	0	0
Average *B* factors (Å^2^)		
Protein	70.3	65.7
Ligand	—	—
Solvent	—	—
R.m.s. deviations		
Bond lengths (Å)	0.002	0.003
Bond angles (°)	0.51	0.55
Ramachandran plot		
Favored (%)	97.1	96.9
Allowed (%)	2.9	3.1
Outliers (%)	0	0

**Table 4:** Cryo-EM data-collection and refinement statistics

	PepT_So2_ embedded in Salipro nanoparticles (PDB entry 6ji1, EMDB ID EMD-9832)			
	0° tilt	30° tilt	40° tilt	50° tilt
Cryo-EM data collection				
TEM	Titan Krios	Talos Arctica		
Accelerating voltage (kV)	300	200		
Camera	Falcon III (counting)	Falcon III (counting)		
Total dose (e Å^−2^)	60	82		
No. of micrographs	864	1275	272	62
No. of particles	130487	229633	49304	9480
3D refinement				
Resolution (Å)	6.7	4.13		
Map-sharpening *B* factor (Å^2^)	−200	−147		
Fourier shell correlation criterion	0.143	0.143		
Particles used in final 3D refinement	3415	43172		
Coordinate refinement and validation				
R.m.s. deviations				
Bond lengths (Å)		0.010		
Bond angles (°)		1.225		
Ramachandran plot				
Favored (%)		83.63		
Allowed (%)		11.06		
Outliers (%)		5.31		
*MolProbity* score		2.09		
Clashscore (all-atom)		7.36		
