# Supplementary material for: Structural basis for oligomerization of the prokaryotic peptide transporter PepTSo2
Source: Acta Crystallogr F Struct Biol Commun. 2019 Apr 24;75(Pt 5):348–58. doi: 10.1107/S2053230X19003546 (PMC6497099; doi:10.1107/S2053230X19003546)
Supplement: Supplementary file 1 [file f-75-00348-sup1.pdf]

STRUCTURAL BIOLOGY COMMUNICATIONS

Volume 75 (2019)

Supporting information for article:

Structural basis for oligomerization of the prokaryotic peptide transporter PepTSo2

Reina Nagamura, Masahiro Fukuda, Akihiro Kawamoto, Kyoko Matoba, Naoshi Dohmae, Ryuichiro Ishitani, Junichi Takagi and Osamu Nureki

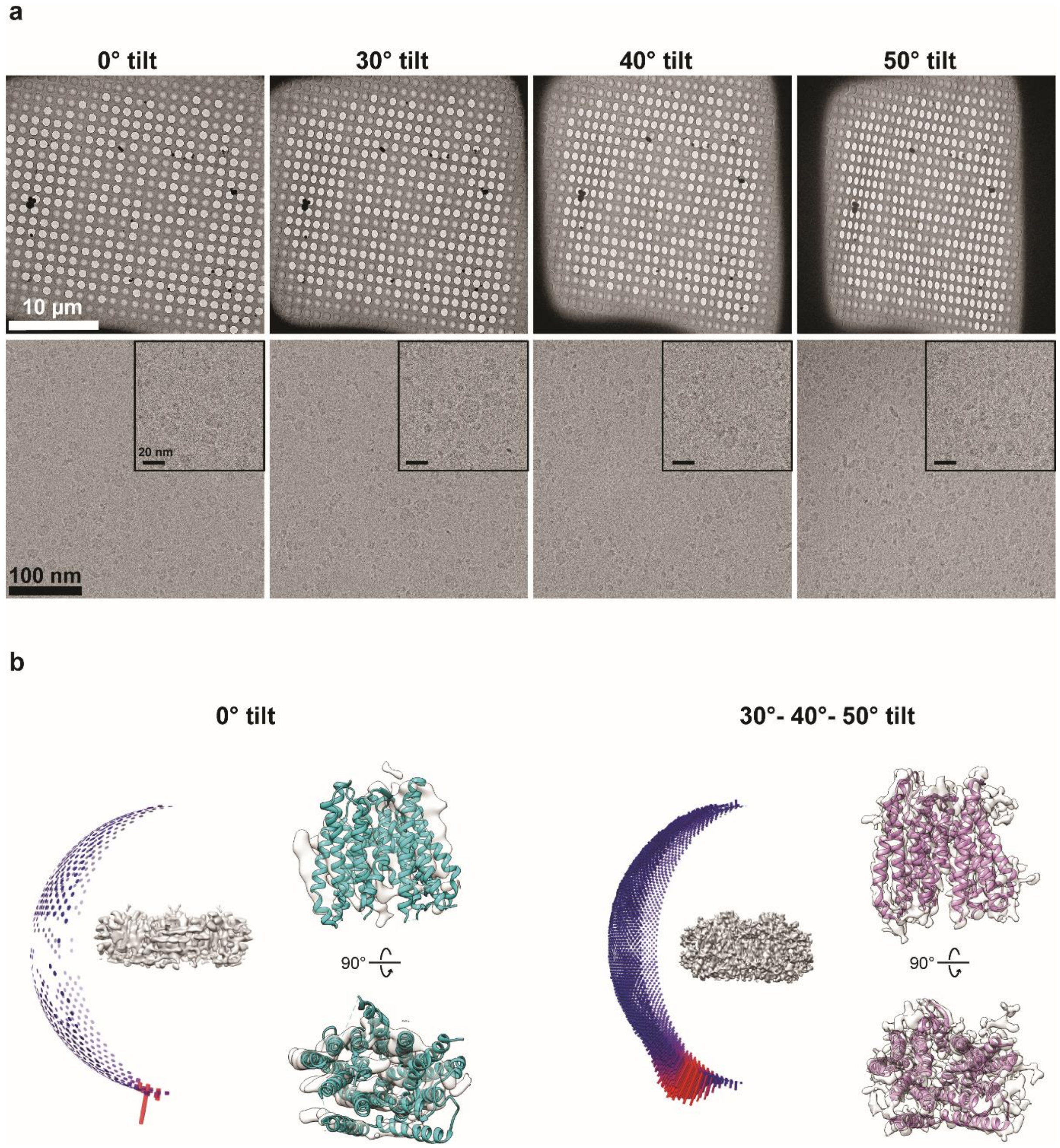

Figure S1 Per-tilt analysis of PepT$_{So2}$. (a) Cryo-EM datasets were collected untilted, or at tilts of 30°, 40°, and 50°. The grid square images of untilted, or 30° tilt, 40° tilt, and 50° tilt are shown in the upper row. Raw EM images of untilted, or 30° tilt, 40° tilt, and 50° tilt are shown in the bottom row. Magnified images of PepT$_{So2}$ are shown in the insets. (b) Euler angle distributions and 3D density maps of the monomeric PepT$_{So2}$ collected by the untilted or tilted method. The crystal structure determined in this study was fitted into each cryo-EM map.

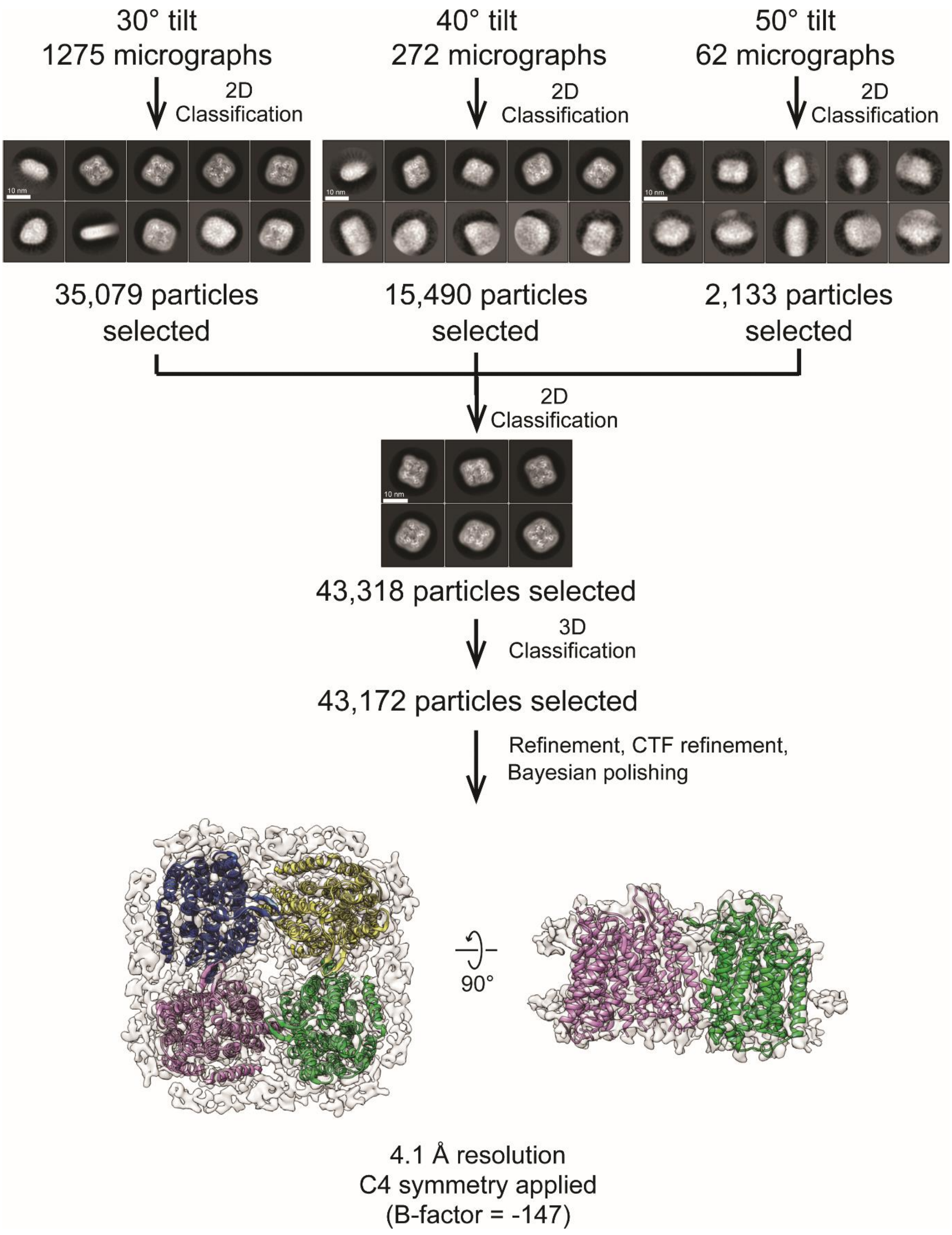
30° tilt
1275 micrographs
2D Classification
40° tilt
272 micrographs
2D Classification
50° tilt
62 micrographs
2D Classification
10 nm
35,079 particles selected
15,490 particles selected
2,133 particles selected
2D Classification
43,318 particles selected
3D Classification
43,172 particles selected
Refinement, CTF refinement, Bayesian polishing
90°
4.1 Å resolution
C4 symmetry applied
(B-factor = -147)

Figure S2 Workflow of cryo-EM image processing. The workflow diagram illustrates the basic strategy practiced in this study. After good particles were selected from the 2D classification at each tilt angle, 43,318 good particles were selected to make the initial 3D model of the $PepT_{So2}$ tetramer and to calculate the 3D classification. Subsequently, 13,644 particles were used for the final 3D auto-refinement, CTF refinement, and Bayesian polishing, yielding a final map at 4.1 Å resolution by gold-standard FSC (Table 4).

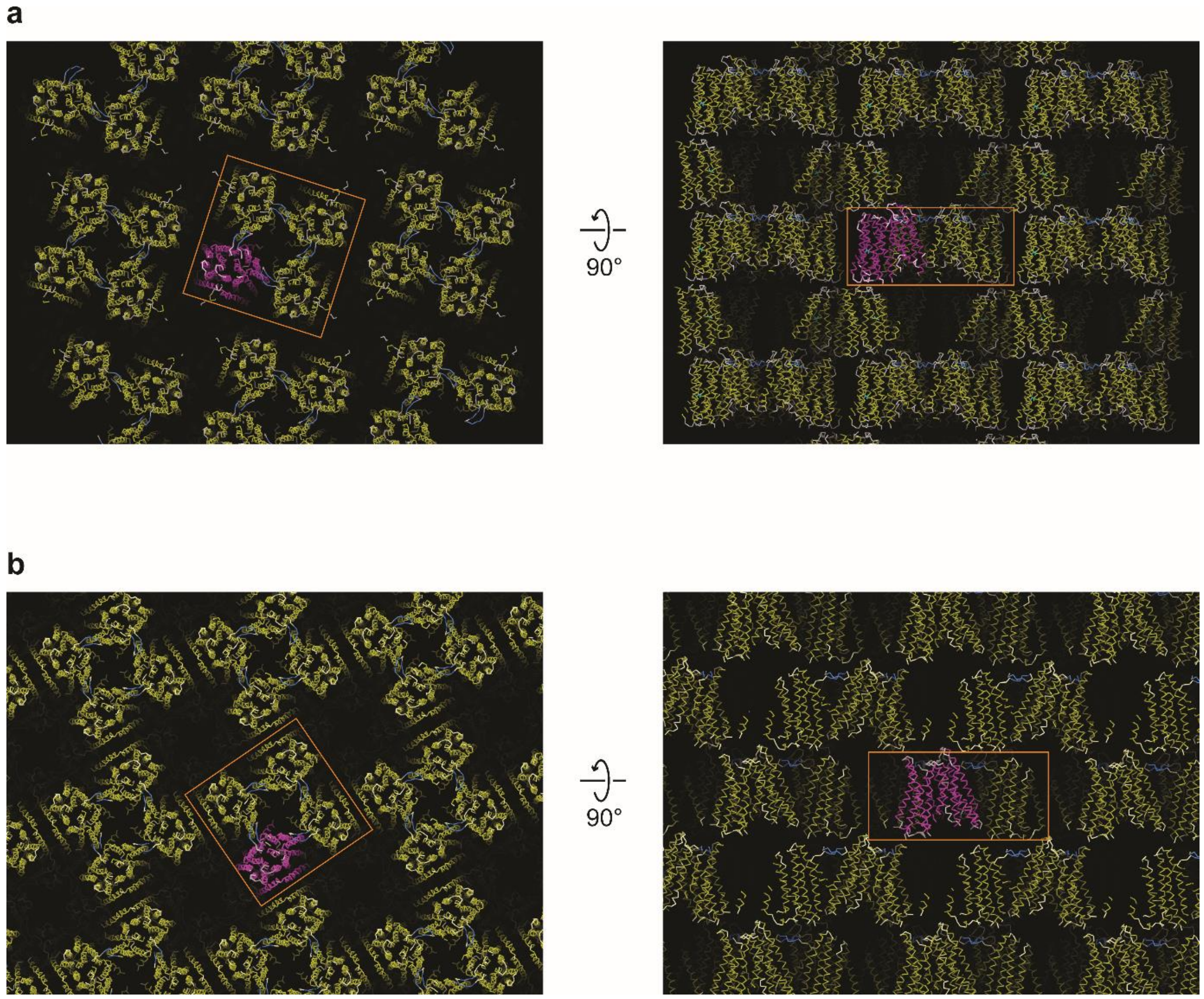

Figure S3 Crystal packings of PepT$_{So2}$ of (a) Form A and (b) Form B, respectively, as viewed from perpendicular to the bilayer and within the lipid bilayer. The PepT$_{So2}$ protomer molecule in the asymmetric unit in each crystal form are colored in pink. The approximate regions of PepT$_{So2}$ tetramer that contains this protomer are indicated in orange rectangles.

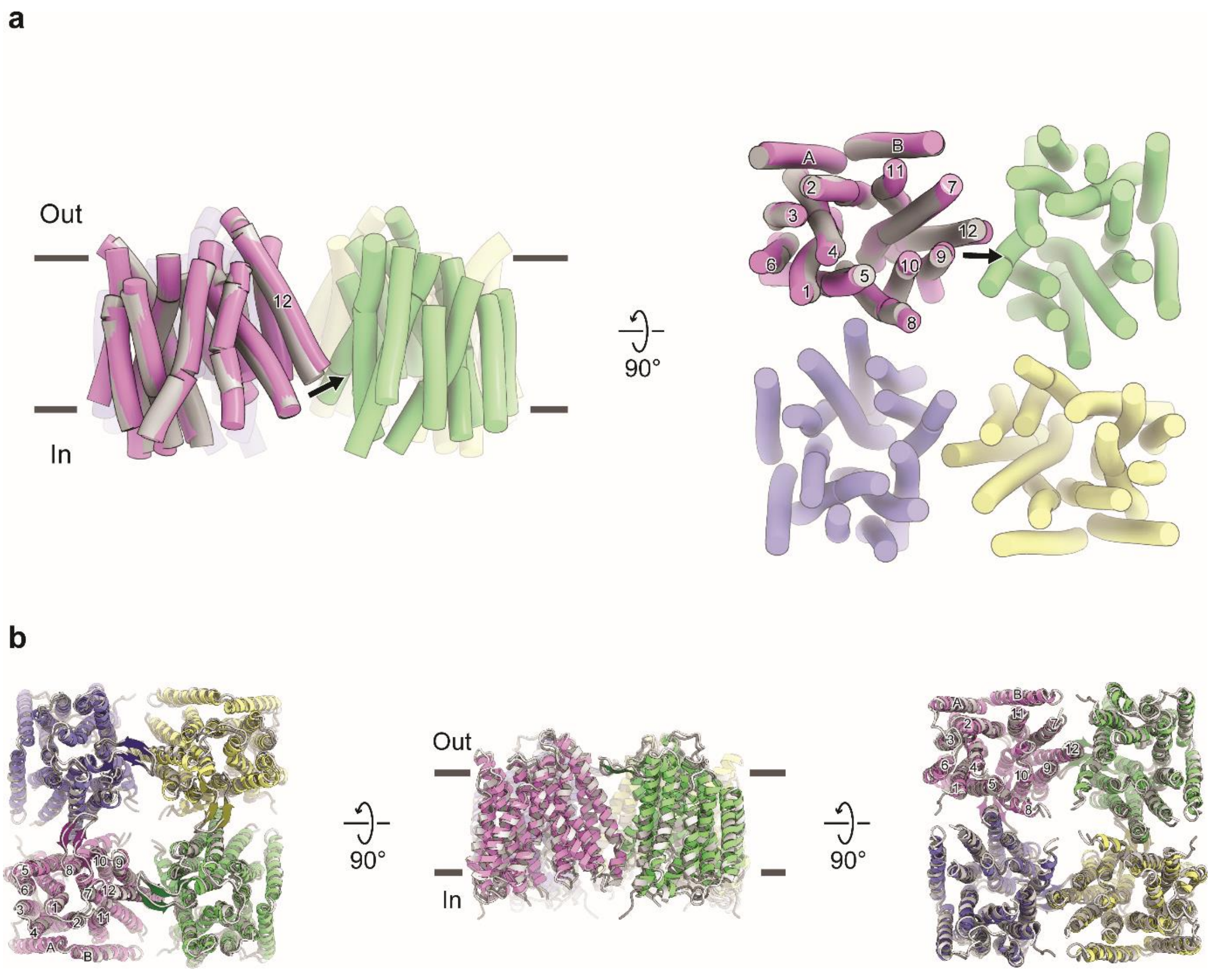

Figure S4 Molecular superposition. (a) Structural comparison of the present tetrameric PepT$_{So2}$ structure (Form B) with the same color scheme as in Fig. 4 and the previously reported dimeric structure colored in grey (PDB accession code: 4LEP). A protomer molecule of the dimeric structure is shown. Sliding motion of H12 is indicated by the black arrow. Two additional helices HA and HB are omitted for clarity in the left panel. The TM helices are shown in cylinder representation and the loop regions between the TM helices are omitted for clarity. (b) Structural comparison of the crystal structure of tetrameric PepT$_{So2}$ (Form B) with the same colour scheme as in Fig. 4 and the cryo-EM atomic model colored in grey.
